# Exploratory analysis of tumor budding density in resected synchronous and metachronous colorectal liver metastases

**DOI:** 10.3389/fonc.2026.1901885

**Published:** 2026-07-15

**Authors:** Nemanja Petrović, Biljana Kukić, Nenad Šolajić, Nemanja Maletin, Ivana Kolarov Bjelobrk, Jelena Radić, Aleksandar Đermanović, Ivan Nikolić

**Affiliations:** 1 Faculty of Medicine Novi Sad, University of Novi Sad, Novi Sad, Serbia; 2 Clinic of Internal Oncology, Oncology Institute of Vojvodina, Sremska Kamenica, Serbia

**Keywords:** colorectal cancer, colorectal liver metastases, histopathological response, metachronous metastases, prognosis, survival, synchronous metastases, tumor budding

## Abstract

**Background:**

Tumor budding is an adverse histopathological feature in colorectal cancer, but its role in colorectal liver metastases (CRLM) remains insufficiently defined. This study explored the association between tumor budding density and clinical outcomes in resected synchronous and metachronous CRLM.

**Methods:**

This single-center ambispective cohort included 77 patients who underwent R0 resection of CRLM between 2007 and 2017. Tumor budding density was assessed in liver metastases and classified as low (0–9 buds per field) or high (≥10 buds per field). Outcomes were analyzed according to tumor budding density, metastatic timing, and preoperative regimen.

**Results:**

Forty-one patients had synchronous and 36 had metachronous CRLM. Overall, 64 patients received preoperative systemic therapy, including FOLFOX4 in 38 and FOLFOX4 plus bevacizumab in 26. Low tumor budding density was associated with better 2-year overall survival in synchronous CRLM, both in the pooled cohort (95.2% vs. 65.0%, p = 0.015) and in patients treated with FOLFOX4 (90.9% vs. 55.6%, p = 0.049) or FOLFOX4 plus bevacizumab (100.0% vs. 66.7%, p = 0.048). No significant association was observed in metachronous CRLM. Kaplan-Meier analyses of time to progression and 5-year overall survival did not show significant differences by tumor budding density.

**Conclusion:**

Tumor budding density showed an exploratory association with 2-year overall survival in patients with synchronous resected colorectal liver metastases, but this association was not consistently observed in metachronous disease or in long-term time-to-event analyses. These findings suggest that tumor budding may reflect aggressive metastatic biology, but larger standardized studies are required before it can be considered an independent prognostic marker.

## Introduction

Colorectal cancer (CRC) remains a major global health burden and is among the most frequently diagnosed malignancies worldwide. According to the most recent GLOBOCAN estimates, colorectal cancer is the third most commonly diagnosed cancer and the second leading cause of cancer-related death globally, underscoring the persistent need for better risk stratification across all stages of disease (1). The liver is the dominant site of distant spread in CRC, and CRLM account for a substantial proportion of disease-related mortality. Although modern systemic therapy, improved imaging, multidisciplinary treatment planning, and advances in hepatic surgery have expanded the pool of patients eligible for potentially curative treatment, outcomes after resection remain heterogeneous, even among patients with apparently limited liver-only disease (2–4).

In contemporary practice, the management of CRLM depends on careful integration of oncologic, surgical, radiologic, and pathological variables. Current ESMO guidance emphasizes that treatment decisions in metastatic CRC should be individualized according to resectability status, disease distribution, molecular profile, treatment response, and patient fitness (2). However, classical clinicopathological factors such as number and size of metastases, timing of metastatic presentation, margin status, and radiologic response still provide only an incomplete estimate of biological aggressiveness (2, 4). This is particularly relevant in patients undergoing resection after preoperative chemotherapy, in whom morphologic regression, residual viable tumor burden, and microscopic invasive behavior may better reflect intrinsic tumor biology than conventional staging variables alone (2, 4, 5).

The timing of liver metastatic spread has long been considered clinically important. Synchronous and metachronous CRLM may differ in tumor biology, disease kinetics, therapeutic strategy, and prognosis, although the magnitude and consistency of these differences vary across cohorts (6, 7). Synchronous disease is often associated with greater initial complexity of management and may reflect a more aggressive phenotype, whereas metachronous disease can represent a more indolent clinical course in selected patients (6, 7). Nevertheless, timing alone is not sufficient for robust prognostic discrimination after liver resection, and there is continuing interest in tissue-based biomarkers that could refine postoperative risk assessment within these clinically defined subgroups (4, 7).

Tumor budding, defined as isolated single tumor cells or small clusters of up to four tumor cells at the invasive front, is now widely recognized as an adverse histopathological feature in primary colorectal carcinoma (8, 9). Biologically, tumor budding is closely linked to epithelial-mesenchymal transition, local invasion, and metastatic competence, and in nonmetastatic CRC it has been associated with nodal involvement, recurrence risk, and inferior survival (8–10). The International Tumor Budding Consensus Conference (ITBCC) established a standardized framework for assessment and reporting of tumor budding in colorectal cancer, which greatly improved reproducibility and facilitated its clinical adoption in primary tumors (8). However, despite its established relevance in the primary setting, the significance of tumor budding in metastatic lesions, particularly resected liver metastases, remains far less well defined (8–10).

In parallel, histopathological regression after preoperative chemotherapy has emerged as another potentially meaningful marker in CRLM. The tumor regression grading system proposed by Rubbia-Brandt and subsequent related approaches have shown that the extent of fibrosis and residual viable tumor within resected liver metastases correlates with oncologic outcome after systemic treatment (5, 11–13). Histological response may therefore capture treatment sensitivity more directly than radiologic criteria alone, particularly in lesions that demonstrate morphologic change without complete disappearance on imaging (5, 11, 12). Yet, the relationship between invasive microscopic patterns such as tumor budding and post-treatment histopathological regression in CRLM has not been sufficiently clarified. This gap is clinically relevant because it addresses two complementary dimensions of tumor behavior: intrinsic invasiveness and therapeutic susceptibility (4, 5, 11–13).

Against this background, the present study was designed to evaluate the therapeutic and prognostic significance of tumor budding density in resected synchronous and metachronous colorectal liver metastases. Specifically, we aimed to assess whether tumor budding density is associated with histopathological response to preoperative therapy, whether it differs in prognostic relevance according to metastatic timing, and whether it may identify patients at increased risk of poor short-term survival despite R0 liver resection. By focusing on a cohort of patients with liver-limited metastatic CRC treated with FOLFOX4, with or without bevacizumab, and assessed using both histopathological regression and survival endpoints, this study addresses a clinically relevant and still insufficiently explored pathological marker in CRLM.

## Methods

### Study design and setting

This study was designed as a single-center ambispective cohort study, combining retrospective identification of eligible cases with prospective-style standardized pathological reassessment of archived specimens. The study included adult patients with CRLM who underwent hepatic resection at the Institute of Oncology of Vojvodina, Serbia, between May 1, 2007 and June 1, 2017. The primary objective was to explore the association between tumor budding density and survival outcomes in resected synchronous and metachronous CRLM, with secondary exploratory analyses of histopathological response after preoperative treatment.

### Patients and eligibility criteria

A total of 120 patients with histologically confirmed colorectal adenocarcinoma metastatic to the liver were screened. Of these, 77 fulfilled the predefined inclusion criteria and were included in the analysis. Eligible patients were required to be older than 18 years, to have liver-limited metastatic colorectal cancer confirmed histopathologically, to have undergone R0 resection of liver metastases, and to have adequate clinicopathological follow-up data available in the institutional hospital information system. Patients with incomplete clinical follow-up data were excluded. The final cohort comprised 41 patients with synchronous metastatic disease and 36 with metachronous metastatic disease. Overall, 64 patients received preoperative systemic therapy, whereas 13 underwent initial surgery for metastatic disease. Two-year overall survival was selected as the primary survival endpoint because the study aimed to identify early postoperative survival differences associated with aggressive metastatic histology in a relatively small resected CRLM cohort.

### Definition of synchronous and metachronous disease

Patients were categorized according to the timing of liver metastatic disease relative to diagnosis and treatment of the primary colorectal tumor. Synchronous CRLM was defined as liver metastases detected before, at the time of, or within 6 months of diagnosis of the primary colorectal carcinoma. Metachronous CRLM was defined as liver metastases detected more than 6 months after diagnosis or surgery of the primary tumor. A 6-month cutoff was used, in line with commonly applied definitions, although some studies have used a 12-month threshold.

### Clinical data collection

Clinical and pathological data were retrieved from the hospital information system of the Institute of Oncology of Vojvodina, including pathology reports, hospitalization records, outpatient follow-up notes, and multidisciplinary tumor board documentation. Extracted variables included age at diagnosis, sex, primary tumor sidedness, timing of metastatic disease, treatment modality, radiologic response, histopathological response, disease progression, and survival status. The cohort represented liver-limited stage IV disease.

### Treatment groups

Among patients who received preoperative treatment, systemic therapy consisted of FOLFOX4 alone or FOLFOX4 combined with bevacizumab. In the analyzed treated subset, 38 patients received FOLFOX4 and 26 received FOLFOX4 plus bevacizumab. Patients with metachronous disease who underwent upfront liver surgery without preoperative systemic therapy were retained in the overall cohort for descriptive and exploratory analyses, but regimen-specific response analyses primarily focused on the pretreated population. Treatment allocation reflected routine clinical decision-making rather than randomization. In routine multidisciplinary practice at our institution, patients with clearly resectable liver metastases were generally treated with FOLFOX4, whereas those considered potentially resectable were more likely to receive FOLFOX4 plus bevacizumab.

### Pathology material and tissue processing

After case selection, archived histological slides and paraffin-embedded tissue blocks from resected liver metastases were reviewed. Tissue specimens had been fixed in formalin and embedded in paraffin according to standard pathology protocols. Sections 4 µm thick were prepared from representative tumor areas, including the invasive front, and stained with hematoxylin and eosin for routine histological evaluation. In addition, immunohistochemical staining was performed using an anti-cytokeratin 20 (CK20) antibody to facilitate identification of tumor buds in challenging cases.

### Immunohistochemistry

For immunohistochemical analysis, paraffin sections were deparaffinized and endogenous peroxidase activity was blocked using 3% hydrogen peroxide for 5 minutes. Antigen retrieval was performed in 10 mM citrate buffer (0.1 M citric acid and 0.1 M sodium citrate, pH 6.0) in a microwave oven, twice for 10 minutes each, followed by rinsing in deionized water. Sections were incubated for 30 minutes at room temperature with anti-CK20 antibody (DAKO, Denmark), followed by a 30-minute incubation with streptavidin-peroxidase complex. 3-amino-9-ethylcarbazole was used as the chromogenic substrate, and hematoxylin was applied as counterstain. Between incubation steps, samples were washed in tris-buffered saline (0.05 M, pH 7.6). Immunohistochemical assessment was performed by light microscopy using qualitative and semiquantitative evaluation with appropriate controls.

### Assessment of tumor budding

Tumor budding was assessed by two pathologists who were blinded to clinical outcomes and treatment response data. Discordant cases were reviewed jointly to reach consensus. Tumor budding density was assessed microscopically on resected liver metastasis specimens. After scanning the invasive front, the hotspot area with the highest density of tumor buds was selected at ×200 magnification using a 20× objective. In accordance with the approach cited in the dissertation from Ueno et al., tumors were categorized into two groups: low TB density (0–9 buds per field) and high TB density (≥10 buds per field). Tumor buds were defined morphologically as isolated single tumor cells or small clusters composed of up to four dedifferentiated tumor cells detached from the main tumor body. When required, CK20 immunostaining was used to support bud identification. In patients with multiple liver metastases, tumor budding assessment was performed on the largest available metastatic lesion with a clearly identifiable invasive front.

### Histopathological response assessment

Histopathological response to preoperative systemic treatment was evaluated using the modified tumor regression grade (mTRG) system for colorectal liver metastases. The modified TRG system was based on previously published criteria, with infarct-like necrosis considered equivalent to fibrosis. For key comparative analyses, response was further dichotomized into favorable response (mTRG 1–2) and unfavorable response (mTRG 3–5), or alternatively poor response defined as mTRG 3–5, depending on the specific analysis.

### Radiologic response assessment

Radiologic response to preoperative treatment was assessed using RECIST criteria on cross-sectional imaging performed before surgery. In the dissertation, histopathological regression according to mTRG was compared with radiologic response according to RECIST. For analytic purposes, radiologic response categories were also grouped, with complete response (CR) and partial response (PR) considered favorable radiologic response, while stable disease (SD) and progression were analyzed separately where appropriate. Only patients with sufficient disease control to proceed to liver resection were included in the pathological study cohort.

### Outcome measures

The primary endpoint was 2-year overall survival after hepatic resection. Secondary endpoints included postoperative disease progression, time to progression, 5-year overall survival, histopathological response according to mTRG, and radiologic response according to RECIST. Disease progression was analyzed both as a binary postoperative event and, where time-to-event data were available, as time to progression. The dissertation also included exploratory long-term analyses of 5-year overall survival and Kaplan-Meier curves for progression and survival according to metastatic timing, treatment regimen, and TB density, although these analyses were limited by sample size and incomplete long-term follow-up in some subgroups.

### Ethics statement

This study was conducted in accordance with all principles of the Declaration of Helsinki and was approved by the Ethics Committee of the Oncology Institute of Vojvodina, Sremska Kamenica, Serbia, on December 23, 2016, approval number 4/4-4403/2-17. All patient data were handled confidentially and anonymized before analysis in accordance with institutional ethical requirements.

### Statistical analysis

Continuous variables were summarized as mean ± standard deviation (SD) or median with range, as appropriate, whereas categorical variables were presented as counts and percentages. Comparisons between two groups were performed using Student’s t-test or the Mann-Whitney U test, depending on data distribution. Categorical variables were compared using the chi-square test or Fisher’s exact test, as appropriate. Overall survival (OS) was defined as the time from liver resection to death from any cause or last follow-up. Time to progression (TTP) was defined as the time from liver resection to documented disease progression or last follow-up. Survival curves were estimated using the Kaplan-Meier method and compared with the log-rank test. To identify factors associated with 2-year overall survival, univariable logistic regression analysis was used, with results presented as odds ratios (ORs) and 95% CIs. All statistical tests were two-sided, and a p value < 0.05 was considered statistically significant. Statistical analyses were performed using SPSS software. Because of the exploratory nature of the study and the limited sample size, subgroup analyses were interpreted descriptively. No adjustment for multiple comparisons was applied; therefore, p values from subgroup analyses should be considered hypothesis-generating rather than confirmatory.

## Results

A total of 77 patients with resected colorectal liver metastases were included, of whom 41 (53.2%) had synchronous and 36 (46.8%) metachronous metastatic disease. The mean age at diagnosis was 61.92 ± 9.97 years, and patients with metachronous metastases were significantly older than those with synchronous disease (65.19 ± 8.17 vs 59.05 ± 10.60 years, p = 0.006). Most primary tumors were left-sided (81.8%). Overall, 64 patients (83.1%) received preoperative systemic therapy, including 38 treated with FOLFOX4 and 26 with FOLFOX4 plus bevacizumab (**Table 1**).

**Table 1:** Baseline clinicopathological characteristics of the study cohort.

Characteristic	Overall (N = 77)	Synchronous CRLM (n = 41)	Metachronous CRLM (n = 36)	p value
Age at diagnosis, years, mean ± SD	61.92 ± 9.97	59.05 ± 10.60	65.19 ± 8.17	0.006
Median age, years (range)	63 (37-77)	NR	NR	–
Male sex, n (%)	45 (58.4)	22 (53.7)	23 (63.9)	0.363
Female sex, n (%)	32 (41.6)	19 (46.3)	13 (36.1)	
Primary tumor location, n (%)				0.788
Left-sided colon	63 (81.8)	34 (82.9)	29 (80.6)	
Right-sided colon	14 (18.2)	7 (17.1)	7 (19.4)	
Initially operated metastatic disease, n (%)	13 (16.9)	3 (7.3)	10 (27.8)	NR
Received preoperative systemic therapy, n (%)	64 (83.1)	38 (92.7)	26 (72.2)	NR
Preoperative regimen among treated patients, n (%)				0.124
FOLFOX4	38/64 (59.4)	20/38 (52.6)	18/26 (69.2)	
FOLFOX4 + bevacizumab	26/64 (40.6)	18/38 (47.4)	8/26 (30.8)	
Tumor budding density, n (%)				–
Low TB (0–9)	34 (44.2)	21 (51.2)	13 (36.1)	
High TB (≥10)	43 (55.8)	20 (48.8)	23 (63.9)	
Histopathological response, n (%)				–
mTRG 1–2	23 (29.9)	12 (29.3)	11 (30.6)	
mTRG 3–5	44 (57.1)	23 (56.1)	21 (58.3)	
Not assessable/missing in pooled summary	10 (13.0)	6 (14.6)	4 (11.1)	
2-year overall survival, n (%)	61 (79.2)	33 (80.5)	28 (77.8)	NR

CRLM, colorectal liver metastases; SD, standard deviation; TB, tumor budding; mTRG, modified tumor regression grade; NR, not reported. Percentages for treatment regimens were calculated among patients who received preoperative systemic therapy.

When outcomes were analyzed according to tumor budding density, low tumor budding was associated with better 2-year overall survival in patients with synchronous colorectal liver metastases, both in the pooled synchronous cohort and in the subgroup treated with FOLFOX4, with a similar pattern observed in the FOLFOX4 plus bevacizumab subgroup. By contrast, no statistically significant association between tumor budding density and 2-year overall survival was observed in metachronous metastases. Histopathological response and progression rates also tended to be less favorable in patients with high tumor budding, particularly in synchronous disease, although these differences did not consistently reach statistical significance (**Table 2**).

**Table 2:** Two-year survival, progression, radiologic response, and histopathological response according to tumor budding density, metastatic timing, and preoperative regimen.

Metastatic setting	Preoperative regimen	Outcome	Low TB, n/N (%)	High TB, n/N (%)	p value
Synchronous CRLM	Pooled	2-year overall survival	20/21 (95.2)	13/20 (65.0)	0.015
Synchronous CRLM	Pooled	Progression after liver surgery	11/21 (52.4)	14/20 (70.0)	0.133
Synchronous CRLM	Pooled	Worse histopathological response (mTRG 3–5)	8/16 (50.0)	15/19 (78.9)	0.072
Synchronous CRLM	Pooled	Favorable radiologic response (CR/PR)	12/21 (57.1)	9/20 (45.0)	NS
Synchronous CRLM	FOLFOX4	2-year overall survival	10/11 (90.9)	5/9 (55.6)	0.049
Synchronous CRLM	FOLFOX4	Progression after liver surgery	5/11 (45.5)	6/9 (66.7)	0.142
Synchronous CRLM	FOLFOX4	Worse histopathological response (mTRG 3–5)	5/8 (62.5)	8/9 (88.9)	0.200
Synchronous CRLM	FOLFOX4	Favorable radiologic response (CR/PR)	6/11 (54.5)	4/9 (44.4)	0.653
Synchronous CRLM	FOLFOX4 + bevacizumab	2-year overall survival	9/9 (100.0)	6/9 (66.7)	0.048
Synchronous CRLM	FOLFOX4 + bevacizumab	Progression after liver surgery	5/9 (55.6)	8/9 (88.9)	0.114
Synchronous CRLM	FOLFOX4 + bevacizumab	Worse histopathological response (mTRG 3–5)	3/8 (37.5)	6/9 (66.7)	0.229
Synchronous CRLM	FOLFOX4 + bevacizumab	Favorable radiologic response (CR/PR)	6/9 (66.7)	5/9 (55.6)	0.629
Metachronous CRLM	Pooled	2-year overall survival	11/13 (84.6)	17/23 (73.9)	0.458
Metachronous CRLM	Pooled	Progression after liver surgery	7/13 (53.8)	14/23 (60.9)	0.346
Metachronous CRLM	Pooled	Worse histopathological response (mTRG 3–5)	8/10 (80.0)	13/18 (72.2)	0.649
Metachronous CRLM	Pooled	Favorable radiologic response (CR/PR)	5/13 (38.5)	8/23 (34.8)	NS
Metachronous CRLM	FOLFOX4	2-year overall survival	7/8 (87.5)	6/10 (60.0)	0.196
Metachronous CRLM	FOLFOX4	Progression after liver surgery	4/8 (50.0)	6/10 (60.0)	0.387
Metachronous CRLM	FOLFOX4	Worse histopathological response (mTRG 3–5)	6/8 (75.0)	7/10 (70.0)	0.814
Metachronous CRLM	FOLFOX4	Favorable radiologic response (CR/PR)	4/8 (50.0)	6/10 (60.0)	0.671
Metachronous CRLM	FOLFOX4 + bevacizumab	2-year overall survival	1/2 (50.0)	4/6 (66.7)	0.673
Metachronous CRLM	FOLFOX4 + bevacizumab	Progression after liver surgery	2/2 (100.0)	5/6 (83.3)	0.537
Metachronous CRLM	FOLFOX4 + bevacizumab	Worse histopathological response (mTRG 3–5)	2/2 (100.0)	4/6 (66.7)	0.346
Metachronous CRLM	FOLFOX4 + bevacizumab	Favorable radiologic response (CR/PR)	1/2 (50.0)	2/6 (33.3)	0.673
Synchronous vs metachronous CRLM	FOLFOX4	Worse histopathological response (mTRG 3–5)	13/17 (76.5) vs 13/18 (72.2)	–	0.774

In univariable logistic regression analyses for 2-year overall survival, high tumor budding density was not a statistically significant predictor in any subgroup, although a borderline association was observed in patients with synchronous metastases treated with FOLFOX4. In the synchronous FOLFOX4 plus bevacizumab subgroup, favorable radiologic response (CR/PR) emerged as a significant protective factor for 2-year overall survival. No other clinicopathological variable showed a consistent significant association with 2-year survival across treatment-defined subgroups (**Table 3**).

**Table 3:** Univariable logistic regression for death within 2 years after liver resection.

Variable	Synchronous CRLM, FOLFOX4 OR (95% CI)	p value	Synchronous CRLM, FOLFOX4 + bevacizumab OR (95% CI)	p value	Metachronous CRLM, FOLFOX4 OR (95% CI)	p value	Metachronous CRLM, FOLFOX4 + bevacizumab OR (95% CI)	p value
Age at diagnosis (years)	0.995 (0.899–1.101)	0.918	1.059 (0.919–1.220)	0.428	1.052 (0.904–1.225)	0.510	0.952 (0.807–1.122)	0.556
Female sex	3.000 (0.372–24.171)	0.302	0.250 (0.021–3.041)	0.277	1.067 (0.129–8.793)	0.952	0.333 (0.017–6.654)	0.472
Right-sided primary tumor	2.667 (0.298–23.858)	0.380	4.333 (0.207–90.847)	0.345	1.375 (0.096–19.643)	0.814	2.000 (0.078–51.593)	0.676
High tumor budding density	8.000 (0.697–91.797)	0.095	4.000 (0.329–48.656)	0.277	4.667 (0.404–53.950)	0.217	0.500 (0.019–12.898)	0.676
mTRG 3–5	1.333 (0.104–17.098)	0.825	2.000 (0.146–27.447)	0.604	0.778 (0.148–21.395)	0.650	0.500 (0.019–12.898)	0.676
CR/PR	0.167 (0.015–1.879)	0.147	0.075 (0.006–0.954)	0.046	0.417 (0.051–3.435)	0.416	0.750 (0.038–14.972)	0.851

OR, odds ratio; CI, confidence interval; CRLM, colorectal liver metastases; TB, tumor budding; mTRG, modified tumor regression grade; CR/PR, complete or partial radiologic response.

Odds ratios above 1 indicate higher odds of death within 2 years after liver resection.

Additional clinicopathological variables are presented in **Supplementary Table 1**. Briefly, 34 patients (44.2%) had liver metastases larger than 3 cm, whereas 43 (55.8%) had metastases smaller than 3 cm. A solitary liver lesion was present in 33 patients (42.9%), 29 (37.7%) had two to three lesions, and 15 (19.5%) had four or more lesions. New liver metastases within the first postoperative year were observed in 18 patients, and extrahepatic spread in 12 patients.

**Figure 1:**
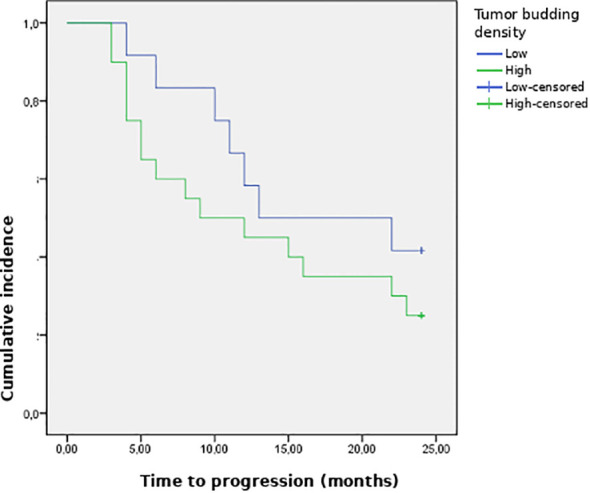
Kaplan–Meier curves for time to progression according to tumor budding density in patients with metachronous CRLM.

Kaplan–Meier analysis of time to disease progression in patients with metachronous colorectal liver metastases showed no statistically significant difference according to tumor budding density (log-rank p = 0.253). Although patients with high tumor budding tended to experience earlier progression, the difference did not reach statistical significance (**Figure 1**).

**Figure 2:**
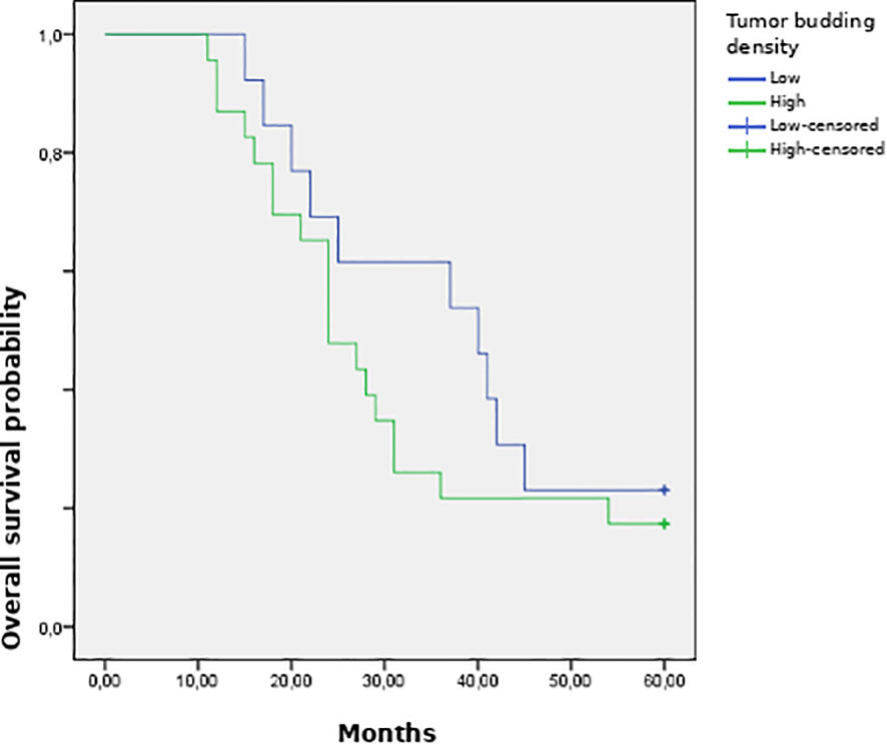
Kaplan–Meier curves for 5-year overall survival according to tumor budding density in patients with metachronous CRLM.

**Figure 2** showed no statistically significant difference in 5-year overall survival between patients with metachronous colorectal liver metastases according to tumor budding density (log-rank p = 0.327). Although patients with high tumor budding density tended to have poorer overall survival, the separation between the curves was not statistically significant.

**Figure 3:**
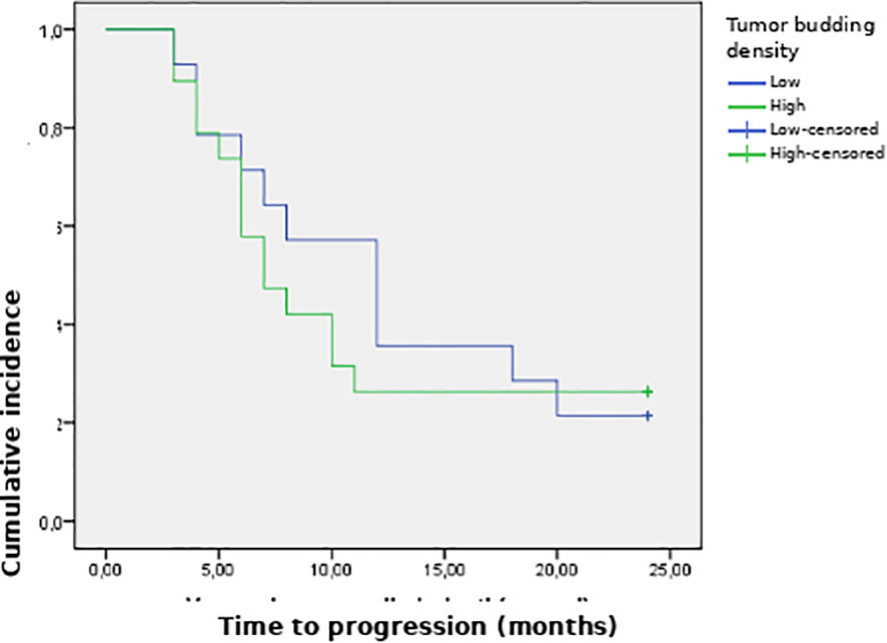
Kaplan–Meier curves for time to progression according to tumor budding density in patients with synchronous CRLM.

**Figure 3** showed no statistically significant difference in time to disease progression between patients with synchronous colorectal liver metastases according to tumor budding density (log-rank p = 0.575). Although patients with high tumor budding density tended to experience earlier progression, the difference between the curves was not statistically significant.

**Figure 4:**
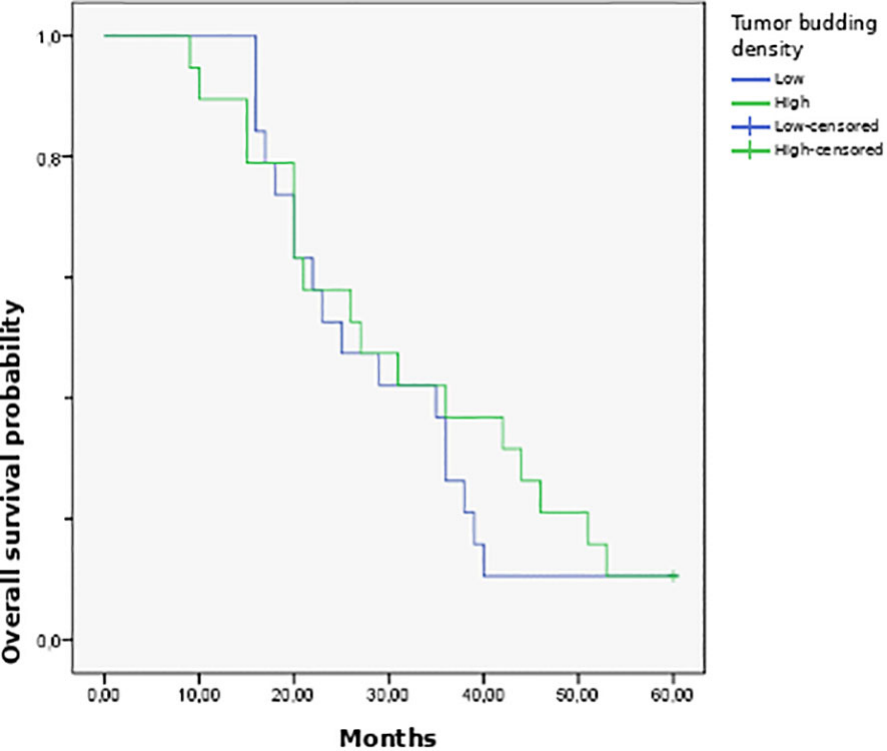
Kaplan–Meier curves for 5-year overall survival according to tumor budding density in patients with synchronous CRLM.

**Figure 4** showed no statistically significant difference in 5-year overall survival between patients with synchronous colorectal liver metastases according to tumor budding density (log-rank p = 0.602). Although patients with high tumor budding density tended to have poorer overall survival, the difference between the survival curves was not statistically significant.

## Discussion

The present exploratory study identified a short-term survival signal associated with tumor budding density in patients undergoing resection of synchronous CRLM. The most consistent finding was the association between low tumor budding density and better 2-year overall survival in patients with synchronous CRLM, including both the pooled synchronous cohort and the subgroups treated with FOLFOX4 with or without bevacizumab. In contrast, this pattern was not clearly observed in metachronous CRLM, and Kaplan-Meier analyses for time to progression and 5-year overall survival did not demonstrate statistically significant differences according to tumor budding density. Therefore, tumor budding in CRLM should be interpreted as a potential marker of aggressive biology rather than as a definitive independent prognostic factor.

Tumor budding is widely recognized as a histological manifestation of tumor cell dissociation, local invasion, and early metastatic competence. The ITBCC recommendations standardized its assessment in colorectal cancer by defining tumor buds as single tumor cells or clusters of up to four tumor cells and by recommending hotspot-based evaluation at the invasive front (8). A large systematic review and meta-analysis showed that tumor budding is strongly associated with lymph node metastasis, recurrence, and cancer-related death in CRC (14, 15). In line with this, Lugli et al. emphasized that tumor budding reflects a broader biological program involving epithelial-mesenchymal transition, extracellular matrix remodeling, stromal activation, and immune escape (9). These mechanisms provide a plausible biological explanation for the poorer short-term survival observed in patients with high tumor budding density in our synchronous CRLM subgroup.

The relevance of tumor budding has also been confirmed in more advanced disease. Qu et al. reported in a meta-analysis of metastatic CRC that high-grade tumor budding was associated with poorer prognosis, suggesting that this marker remains biologically meaningful beyond localized disease (16). In colorectal cancer cohorts, Sarkar et al. further supported the applicability of tumor budding and poorly differentiated clusters as adverse histopathological markers (17). This finding is highly consistent with our data. In our cohort, tumor budding showed a clear association with 2-year survival in synchronous CRLM, but this effect was not uniformly reproduced across all survival and regression analyses. Thus, tumor budding appears to identify an aggressive histological phenotype, but it should probably be integrated with other clinical and pathological variables rather than used alone.

The biological plausibility of our findings is further supported by studies linking tumor budding with the immune microenvironment. Fujiyoshi et al. analyzed 915 colorectal carcinomas and demonstrated that tumor budding was inversely associated with intraepithelial cytotoxic T-cell densities, particularly CD3+CD8+ and CD3+CD8+CD45RO+ memory T cells (18). They also showed that high-grade tumor budding was associated with increased colorectal cancer-specific mortality, independently of molecular features and immune cell densities (18). These findings support the concept that tumor budding represents a tumor-host interface phenomenon: tumor buds reflect invasive pressure, while cytotoxic T cells represent local immune containment. When this balance shifts toward invasion, prognosis worsens.

Translational data further suggest that tumor budding is connected with immune escape and mesenchymal biology. Guil-Luna et al. showed that high-grade tumor budding is associated with the poor-prognosis CMS4 mesenchymal subtype and with upregulation of immune-evasion and inflammatory pathways, including PDL1, TIM-3, NOX2, IDO1, Toll-like receptors, and chemokine-related signaling (19). Similarly, Haddad et al. demonstrated that tumor budding and Immunoscore provide complementary prognostic information in colon cancer, supporting the idea that invasive morphology and immune contexture should be interpreted together (20). Although immune markers were not available in our cohort, these studies provide a strong rationale for future CRLM studies combining tumor budding density with CD8+ T-cell infiltration, macrophage phenotype, tertiary lymphoid structures, and spatial immune features.

The difference between synchronous and metachronous CRLM observed in our study may reflect differences in disease kinetics and biological selection. Synchronous metastases often represent earlier systemic dissemination and may therefore enrich the cohort for tumors with more aggressive invasive potential (21). However, metastatic timing alone should not be overinterpreted. A contemporary cohort study by Johannsen et al. showed that the apparent adverse prognosis of synchronous CRLM may be attenuated after adjustment for tumor burden, molecular characteristics, CEA level, and treatment strategy (22). This supports a more nuanced interpretation of our results: tumor budding may be more informative in synchronous CRLM not because synchronicity itself is causal, but because synchronous disease may represent a clinical context in which invasive tumor biology is more pronounced (23, 24).

The relationship between tumor budding and treatment response remains unresolved. In our study, high tumor budding was numerically associated with less favorable histopathological response in synchronous CRLM, but this association did not consistently reach statistical significance (25). Histopathological response after preoperative chemotherapy is a well-established prognostic feature in CRLM (5, 22), yet it is influenced by several factors, including baseline tumor biology, chemotherapy exposure, timing of surgery, vascular effects of bevacizumab, and heterogeneity between metastases (26). Therefore, tumor budding should not be considered a predictive biomarker for FOLFOX4-based therapy on the basis of the present data, but rather as a candidate marker of invasive and potentially less treatment-sensitive morphology.

An additional point concerns the potential influence of preoperative systemic therapy on tumor budding density. In this cohort, tumor budding was assessed in resected liver metastases after preoperative treatment in most patients, meaning that the observed budding pattern may reflect both intrinsic invasive tumor biology and therapy-induced selection of residual tumor cell populations. Preoperative chemotherapy in CRLM can reduce viable tumor burden and induce fibrosis or other regression-related changes, and histopathological response after chemotherapy has been associated with clinical outcome (5, 11, 12). At the same time, tumor budding represents a marker of tumor cell dissociation, epithelial–mesenchymal transition, and invasive capacity (8, 9). Therefore, residual tumor buds at the invasive front after systemic therapy may represent a treatment-resistant and highly invasive subpopulation. This interpretation is supported by studies in neoadjuvant-treated colorectal cancer showing that tumor budding assessment after preoperative therapy retains prognostic relevance (27). However, because treatment allocation in the present cohort was not randomized and pretreatment biopsy-based tumor budding assessment was not available, this study cannot determine whether preoperative therapy modifies tumor budding density or whether high tumor budding primarily reflects baseline tumor aggressiveness. Future studies comparing pretreatment and post-treatment specimens are needed to clarify whether tumor budding is a stable prognostic marker or a dynamic feature influenced by neoadjuvant treatment.

A more complete pathological risk model for CRLM will probably require integration of tumor budding with other features of the metastatic tumor-liver interface (28, 29). Histopathological growth patterns, particularly desmoplastic and replacement patterns, have emerged as important prognostic and biological markers in liver metastases (12, 30, 31). Updated consensus guidelines emphasize that these growth patterns are linked to vascularization, stromal response, immune infiltration, and treatment response (12, 32). Stremitzer et al. further showed that histopathological growth pattern is associated with immune phenotype and outcome in patients with CRLM (33). More recently, Krzywoń et al. also reported that desmoplastic growth pattern is associated with improved survival in CRLM, reinforcing the importance of the tumor–liver interface in prognostication (34). In this context, tumor budding should be viewed as one component of a broader invasive-front phenotype rather than a stand-alone marker.

Methodological standardization remains a key issue. Conventional tumor budding assessment is practical and clinically understandable, but interobserver variability and the complexity of treated metastatic specimens remain relevant limitations. Äijälä et al. recently evaluated a distance-based tumor budding metric in two independent CRC cohorts and showed that greater tumor bud distance was associated with adverse clinicopathological features and worse cancer-specific survival; however, it did not provide additional prognostic information beyond conventional tumor budding grade (35). This supports the continued use of conventional tumor budding density as the most interpretable metric. At the same time, artificial intelligence-based pathology may improve reproducibility in the future (36, 37). Lobanova et al. reviewed AI-based approaches for tumor budding and tumor microenvironment assessment in CRC and concluded that machine-learning and deep-learning models show promising performance, although methodological heterogeneity still limits immediate clinical translation (38).

This study has several important limitations. First, it was a single-center study with a small cohort, and subgroup analyses were underpowered after stratification by metastatic timing, preoperative regimen, and tumor budding density. Second, treatment allocation was not randomized and was influenced by clinical resectability assessment, creating potential confounding by indication. Third, molecular data, including RAS, BRAF, and MSI/MMR status, were incomplete and could not be incorporated into adjusted prognostic models. Fourth, tumor budding assessment in treated CRLM is less standardized than in primary colorectal cancer, and the cutoff used in this study cannot be directly equated with ITBCC BD categories. Fifth, the main significant association was observed for 2-year overall survival, whereas time-to-progression and 5-year overall survival analyses did not show statistically significant differences. Therefore, the findings should be interpreted as exploratory and require external validation.

## Conclusion

In this exploratory single-center cohort, low tumor budding density was associated with better 2-year overall survival in patients with synchronous resected CRLM. However, this association was not observed in metachronous disease and was not confirmed in long-term time-to-event analyses. Therefore, tumor budding density should be interpreted as a candidate marker of aggressive metastatic biology rather than an established independent prognostic factor. Larger multicenter studies with standardized budding assessment, molecular annotation, and adequate adjustment for clinicopathological confounders are required before clinical implementation.

## Data Availability

The raw data supporting the conclusions of this article will be made available by the authors, without undue reservation.
