# Supplementary material for: Exploratory analysis of tumor budding density in resected synchronous and metachronous colorectal liver metastases
Source: Front Oncol. 2026 Jul 15;16:1901885. doi: 10.3389/fonc.2026.1901885 (PMC13414808; doi:10.3389/fonc.2026.1901885)
Supplement: Supplementary file 1 [file Table1.docx]

| Variable | Overall (N = 77) |
| --- | --- |
| Largest metastasis >3 cm, n (%) | 34 (44.2) |
| Largest metastasis <3 cm, n (%) | 43 (55.8) |
| One liver nodule, n (%) | 33 (42.9) |
| Two to three liver nodules, n (%) | 29 (37.7) |
| Four or more liver nodules, n (%) | 15 (19.5) |
| Interval from end of chemotherapy to surgery >2 months, n (%) | 37 (48.1) |
| Interval from end of chemotherapy to surgery <2 months, n (%) | 27 (35.1) |
| RAS wild-type among tested patients, n | 9 |
| KRAS-mutant among tested patients, n | 12 |
| New liver metastases within 1 year, n | 18 |
| Extrahepatic spread within 1 year, n | 12 |

**Supplementary Table 1. Additional pathological and clinical variables available in the cohort**
